# Comparative effects of structured exercise protocols on depression and anxiety symptoms: a network meta-analysis

**DOI:** 10.3389/fpsyt.2026.1870361

**Published:** 2026-06-08

**Authors:** Zhiyuan Zhang, Fengyun Li, Can Han, Wenkun Song, Sifan Pu

**Affiliations:** 1 School of Physical Education, Xizang Minzu University, Xianyang, Shaanxi, China; 2 School of Physical Education and Training, Xi’an Physical Education University, Xi’an, Shaanxi, China

**Keywords:** anxiety, depression, exercise, randomized controlled trial, network meta-analysis

## Abstract

**Objective:**

To systematically compare the intervention effects of eight common structured exercise modalities on depressive and anxiety symptoms in adults using a network meta-analysis approach, providing evidence-based insights for developing precision exercise prescriptions in mental health.

**Methods:**

A computer search was conducted across PubMed, Web of Science, Embase, and the Cochrane Library databases, covering the period from the database establishment to March 31,2026. Randomized controlled trials comparing different exercise modalities for treating adult depressive or anxiety symptoms were included. Literature quality was assessed using the Cochrane Collaboration’s Bias Risk Assessment Tool (Revised Version), with statistical analyses performed using Stata 19.0 and RevMan 5.4. The standardized mean difference (SMD) was used as the effect measure, consistency was evaluated by the node splitting method, and the cumulative rank-sum area under the curve (SUCRA) was calculated to rank intervention efficacy. Publication bias was assessed and corrected using the trimming method and meta-regression.

**Results:**

A total of 22 studies involving 23 randomized controlled trials with 1,830 participants were included, encompassing eight exercise modalities: yoga, Tai Chi, Pilates, resistance training, aerobic exercise, combined exercise, high-intensity interval training, and moderate-intensity continuous training. Traditional meta-analysis demonstrated that exercise intervention groups showed significantly better improvements in both depressive symptoms (SMD = -0.67,95% CI: -0.97 to-0.37) and anxiety symptoms (SMD = -0.77,95% CI: -1.12 to-0.41) compared to the control groups (both P <0.001). The network meta-analysis results demonstrated that all exercise modalities were effective. In the SUCRA probability ranking, yoga ranked first in both depression (SUCRA = 68.8%) and anxiety (SUCRA = 72.2%) improvement. However, the differences in effect sizes between yoga and moderate-intensity continuous training (MICT) or high-intensity interval training (HIIT) were not statistically significant (all 95% confidence intervals included 0), indicating no clear superiority among the three interventions; thus, this ranking should be regarded as exploratory rather than confirmatory evidence. The top three interventions for depression symptom improvement were yoga, MICT (56.7%), and HIIT (54.6%), while the top three for anxiety symptom improvement were yoga, HIIT (57.4%), and MICT (56.2%). Subgroup analyses revealed no statistically significant moderating effects of intervention duration or age (all P>0.05), although the effect sizes were larger in the elderly group (≥60 years) compared to other age groups. Consistency testing indicated a reliable evidence network (P>0.05). Egger’s test suggested potential publication bias (depression P = 0.017, anxiety P = 0.010), but the meta-analysis did not incorporate missing studies, and meta-regression did not detect small-sample effects; the direction and significance of the effects remained unchanged, rendering the conclusions robust.

**Conclusion:**

Different exercise modalities exhibit beneficial effects on both depression and anxiety symptoms. Yoga, MICT, and HIIT all demonstrated significant potential in alleviating both symptoms; however, the differences in efficacy among the three interventions were not statistically significant, making them all viable prioritized exercise modalities in clinical practice. Clinical selection should be based on a comprehensive evaluation of the patient’s dominant symptom cluster, tolerance, preferences, and safety profile to develop individualized exercise regimens. Since only one study included Pilates, its independent effect remains to be validated.

**Systematic review registration:**

https://www.crd.york.ac.uk/prospero/, identifier.

## Graphical Abstract

**Figure:**
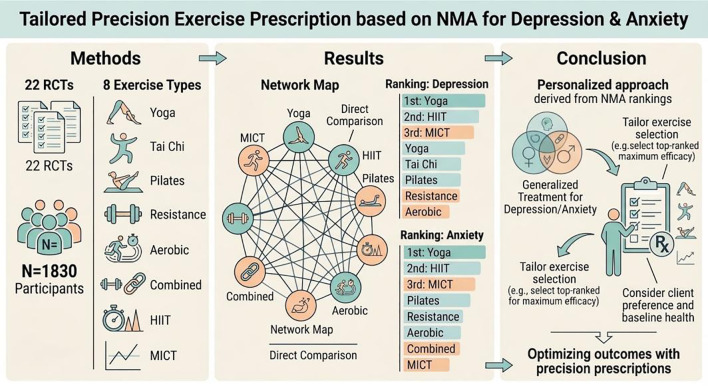
Summary of key findings from the meta-analysis.

## 1 Introduction

Depression and anxiety are highly prevalent mental health disorders worldwide, often co-occurring (1). They severely impair individuals’ cognitive function, emotional regulation, and social adaptation abilities, making them major public health issues (2). According to the World Health Organization, the global prevalence of depressive disorders is approximately 4.4%, while that of anxiety disorders is about 3.6% (3, 4). Current clinical interventions primarily involve pharmacotherapy (5) and psychotherapy (6), but these approaches face limitations such as adverse drug effects, poor patient adherence, and insufficient accessibility of psychotherapy resources.

Against this backdrop, exercise interventions have been extensively studied and demonstrated to provide definitive improvements in mood disorders due to their advantages of safety, cost-effectiveness, ease of implementation, and absence of pharmacological side effects (7–11). However, the efficacy of exercise interventions is modulated by multiple factors, including exercise modalities, intensity, frequency, and duration, with the selection of exercise modalities being one of the key determinants of intervention outcomes in depression (12, 13). Common exercise modalities can be categorized into four major types: aerobic exercise, resistance exercise, mind-body exercises, and mixed exercises, each exhibiting distinct physiological and psychological mechanisms for mood improvement. Recent evidence further highlights that the total volume and intensity of physical activity constitute critical dimensions of health-related benefits, with activities at different intensity levels contributing differently to health outcomes (14). This underscores the clinical necessity for comparative studies that simultaneously evaluate intensity-based exercise modalities (e.g., moderate-intensity continuous training and high-intensity interval training) alongside traditional exercise approaches.

Although traditional meta-analyses have been widely applied in this field, they are mostly limited to direct comparisons or pairwise comparisons between single exercise modalities and conventional care, failing to integrate both direct and indirect comparative evidence from multiple exercise modalities simultaneously, thereby making it difficult to precisely quantify the relative advantages of different exercise modalities (15, 16). Previous systematic reviews have independently demonstrated that meditation-based exercises (e.g., yoga, tai chi) exert beneficial effects on major depressive disorder (17), that mindfulness-based Baduanjin improves depression and anxiety (18), and that tai chi/yoga modulates heart rate variability and perceived stress (19). However, most of these reviews focus on specific families of physical and mental exercises, and there is a lack of network meta-analyses that simultaneously compare physical and mental exercises, aerobic/resistance exercises, and intensity-classified exercise modalities (e.g., HIIT, MICT) within a unified evidence network. Network Meta-Analysis (NMA), as an extension of traditional meta-analysis, enables the construction of evidence networks encompassing multiple interventions, facilitating comprehensive horizontal comparisons and rankings of their efficacy (20, 21) Given the growing importance of personalized and adaptive physical activity interventions, comparative evidence across different exercise modalities is essential for guiding clinical decisions tailored to various symptoms, preferences, baseline physical fitness, and tolerances (22).

Based on this, this study employed a network meta-analysis approach to systematically search for and include randomized controlled trials from abroad comparing different structured exercise interventions for adult depression and anxiety symptoms. The objectives were: (1) to integrate direct and indirect comparative evidence, quantify and rank the relative intervention effects of eight common exercise modalities; (2) to assess the consistency and reliability of the evidence network; and (3) to provide high-quality evidence-based insights for the precise formulation of exercise prescriptions in clinical and community mental health services.

## 2 Research methods

### 2.1 Research design and registration

This study strictly adhered to the Reporting Guidelines for Systematic Reviews and Meta-Analyses (PRISMA 2020) (23), its Extended Version for Network Meta-Analyses (PRISMA-NMA) (24), and the Cochrane Manual for Systematic Reviews (25). As a prospective study, it has been registered on the PROSPERO international systematic review registry (Registration No.: CRD420261359298).

### 2.2 Literature retrieval strategy

The computer system searched four electronic databases—PubMed, Web of Science, Embase, and the Cochrane Library—with search periods spanning from the establishment of each database to March 31,2026. The search employed a combination of subject terms and free-text keywords. Taking PubMed as an example, the search strategy was as follows:

#1 “Exercise”[Mesh] OR “Exercise Therapy”[Mesh] OR aerobic*[tiab] OR resistance[tiab] OR “mind-body”[tiab] OR yoga[tiab] OR “tai chi”[tiab] OR pilates[tiab] OR “high-intensity interval”[tiab] OR HIIT[tiab] OR “moderate-intensity continuous”[tiab] OR MICT[tiab]
#2 “Depression”[Mesh] OR “Anxiety”[Mesh] OR depress*[tiab] OR anxiety[tiab] OR “mood disorder”[tiab]
#3 “Randomized Controlled Trial” [Publication Type] OR “controlled clinical trial”[Publication Type] OR random[tiab] OR RCT[tiab]
#4 #1 AND #2 AND #3

The complete retrieval strategies for other databases are detailed in **Supplementary Table 1**. Additionally, a manual retrospective search was conducted on the literature lists included in the study to identify relevant research that might have been missed through electronic searches. This study only searched the aforementioned English-language databases and did not systematically search Chinese-language databases, which may introduce language bias.

### 2.3 Inclusion and exclusion criteria

#### 2.3.1 Inclusion criteria

(1) Study Type: Published randomized controlled trials (RCTs), with language restricted to English. (2) Study Subjects: Adults aged ≥18 years who were clinically diagnosed (based on DSM-5/ICD-11 criteria) or assessed using standardized scales (e.g., SDS ≥50 points, PHQ-9 ≥10 points, GAD-7 ≥8 points) to exhibit depressive or anxiety symptoms. Excluded individuals with severe somatic diseases (e.g., unstable angina, uncontrolled hypertension), severe mental disorders (e.g., schizophrenia spectrum disorders), or exercise contraindications. (3) Intervention Measures: The trial group received a structured, supervised exercise intervention comprising at least one of yoga, tai chi, Pilates, resistance training, aerobic exercise, combined exercise (combination of ≥2 modalities), high-intensity interval training (HIIT), or moderate-intensity continuous training (MICT). The protocol must specify the intensity, frequency, duration, and cycle length. (4) Control Measures: The control group received routine care, health education, wait-and-see treatment, or maintained daily activities without any structured exercise intervention. (5) Outcome Measures: At least one depression or anxiety-related outcome measure was reported, assessed using standardized scales. Depression assessment tools included SDS, BDI, CES-D, HADS-D, HDRS-17, EPDS, and PHQ-9; anxiety assessment tools included SAS, HAM-A, STAI, BAI, GAD-7, and HADS-A. (6) Intervention Duration: The intervention cycle lasted ≥2 weeks, with sessions ≥1 time per week and each session lasting ≥20 minutes.

#### 2.3.2 Exclusion criteria

(1) Non-RCT studies, reviews, meta-analyses, conference abstracts, case reports, etc.; (2) Literature with incomplete data or unable to extract pre-and post-intervention means ± standard deviations; (3) Studies involving special populations such as children and adolescents; (4) Exercise interventions combined with pharmacotherapy or psychotherapy, where the exercise intervention effect size cannot be isolated; (5) Interventions with unclear protocols or unsupervised, unstructured activities (e.g., merely recommending increased walking).

### 2.4 Literature screening and data extraction

The literature screening and data extraction were independently conducted by two researchers. In case of discrepancies, they were resolved through discussion or consultation with a third-party researcher. The screening process followed these steps: first, duplicate articles were removed using EndNote X9 software; second, obviously irrelevant articles were excluded based on title and abstract reviews; finally, full-text reviews were performed to determine the final included studies.

Data extraction utilized pre-designed standardized Excel sheets, covering the following aspects: (1) basic study information; (2) characteristics of study subjects (sample size, age, diagnostic criteria); (3) details of intervention and control protocols (exercise patterns, intensity, frequency, duration); (4) outcome measures: mean ± standard deviation of depression and anxiety scale scores before and after intervention; (5) methodological information (randomization, concealed assignment, blinding).

### 2.5 Bias risk assessment

Using the Cochrane Risk of Bias Tool (original version), the methodological quality of the 23 included RCTs was evaluated across seven dimensions: random sequence generation, concealed assignment, blinding of participants and investigators, blinding of outcome assessors, data completeness, selective reporting, and other biases. Each dimension was assessed as low risk of bias, high risk of bias, or unknown risk by two independent researchers, with any discrepancies resolved by a third party.

#### 2.5.1 Principles for intervention classification

Due to the conceptual asymmetry among the eight included exercise modalities, the following explanations are provided: Yoga, Tai Chi, and Pilates are defined based on their exercise modalities (physical and mental exercises); High-Intensity Interval Training (HIIT) and Moderate-Intensity Continuous Training (MICT) are categorized according to their intensity distribution characteristics; Resistance exercises and aerobic exercises are classified primarily based on their physiological functional systems; combined exercises represent a combination of multiple modalities. Although this classification approach is not entirely consistent at the conceptual level, it reflects the most common types of structured exercise interventions in current clinical research in this field, facilitating practical application in prescription design. Detailed information on the specific exercise subtypes in each included study, whether they incorporate breathing/meditation/mindfulness components, and supervision status are provided in **Supplementary Table 3**.

### 2.6 Statistical analysis

Statistical analysis was performed using Stata 19.0 and RevMan 5.4 software, with an alpha level of α=0.05. 1) Effect size selection: For continuous outcome data, the standardized mean difference (SMD) and 95% confidence interval (CI) were used as effect sizes; an SMD <0 indicated superior efficacy of the exercise intervention group compared to the control group. 2) Heterogeneity testing: The I² statistic was employed to assess heterogeneity between studies: I² <50% indicated low heterogeneity, warranting pooled analysis using a fixed-effects model; I² ≥50% indicated moderate or high heterogeneity, requiring pooled analysis with a random-effects model. 3) Consistency testing: The node-splitting method was used to evaluate consistency between direct and indirect comparisons; a P-value>0.05 indicated good consistency, justifying the use of a consistency model; otherwise, a non-consistency model was applied. 4) Efficacy ranking: The cumulative rank-sum area under the curve (SUCRA) was calculated, with values ranging from 0% to 100%; higher SUCRA values indicated superior efficacy of the exercise intervention. A cumulative rank-sum probability plot (SUCRA plot) was generated to visually display the ranking probabilities of each intervention. 5) Publication bias assessment: Correction funnel plots, Begg’s test, and Egger’s test were employed to evaluate publication bias, with the more sensitive Egger’s test serving as the definitive criterion; a P-value <0.05 indicated statistically significant publication bias.

### 2.7 Transitivity assessment

The validity of the network meta-analysis relies on sufficient comparability among participants and study contexts across intervention nodes. To this end, this study specifically compared the distributions of key effect-modifying factors—including age range, gender ratio, baseline severity of depression/anxiety, diagnostic type, comorbidity status, medication use rate, intervention duration, frequency, duration per session, supervision ratio, and control type—at each intervention node. Detailed data are summarized in **Supplementary Table 2**. Despite clinical heterogeneity among studies, the node splitting test revealed no inconsistency between direct and indirect evidence, suggesting that the transitivity assumption is valid within the current evidence network.

## 3 Research results

### 3.1 Literature screening process and results

**Figure 1:**
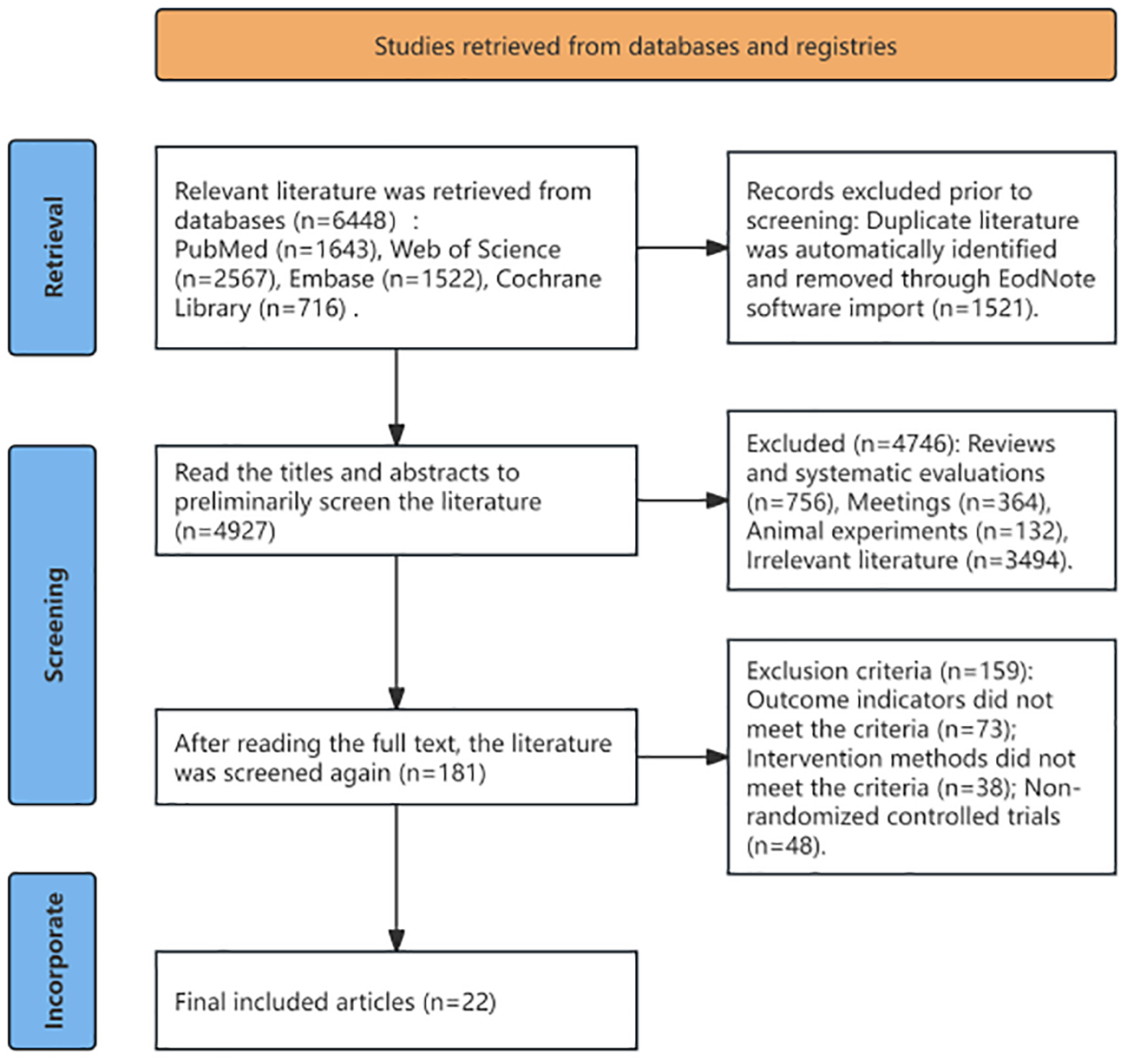
Flowchart of literature screening.

The initial screening yielded a total of 6,448 literature articles. After deduplication using EndNote, 1,521 articles were excluded, leaving 4,927 articles. Following preliminary screening based on titles and abstracts, 4,746 irrelevant articles were excluded. Subsequently, 181 full-text articles were reviewed for further screening, ultimately resulting in the inclusion of 22 RCT articles meeting the criteria. The detailed screening process is illustrated in **Figure 1**.

### 3.2 Basic characteristics of the study participants

The 22 included randomized controlled trials (RCTs) were published between 2013 and 2026, spanning 13 countries/regions and involving a total of 1,830 subjects (918 in the intervention group and 912 in the control group). The average age range of the subjects was 18.8 to 71.2 years.

The intervention modalities included yoga (10 sessions), Tai Chi (3 sessions), Pilates (1 session), resistance training (3 sessions), aerobic exercise (1 session), compound exercises (3 sessions), HIIT (2 sessions), and MICT (2 sessions). The intervention duration ranged from 2 to 24 weeks, with frequencies of 1 to 14 sessions per week and individual session durations of 20 to 60 minutes. All studies used no-intervention exercise (standard care, health education, or maintenance of daily activities) as the primary control. Notably, the study by Kwok et al. (2019) provided a direct head-to-head comparison between yoga and resistance training, forming a critical closed loop in the evidence network. Detailed characteristics of the included studies are presented in **Table 1**.

**Table 1:** Basic characteristics of the included studies.

Research literature (year)	Country	Age (years)	Number of people (T/C)	Intervention study	Control measures	Frequency (per time/week)	Duration ( Zhou )	Depression scale	Anxiety scale
Xu K et al. (2026) (26)	China	20.0 ± 2.6	45/50	TC	NI	2	8	SDS	SAS
Sunil S Y et al. (2024) (27)	India	47.9 ± 11.4	32/31	YG	NI	14	2	DASS-21	DASS-21
Yu et al.a (2023) (28)	Hong Kong, China	63.5 ± 5.3	10/10	MICT	NI	1	12	BDI	GAD-7
Yu et al.b (2023) (28)	Hong Kong, China	63.5 ± 5.3	10/10	HIIT	NI	3	12	BDI	GAD-7
Sun J et al. (2024) (29)	China	18.8 ± 1.1	47/41	TC	NI	3	16	SDS	HAM-A
Whitworth et al.a (2019) (30)	America	33.0 ± 13.3	9/10	RE	NI	9	3	CES-D	STAI
Whitworth et al.b (2019) (31)	America	29.1 ± 7.4	15/15	RE	NI	9	3	CES-D	STAI
Udatha T R et al. (2020) (32)	India	41.8 ± 11.1	30/33	AE	NI	3	8	BDI	HAM-A
Papp M.E et al. (2019) (33)	Sweden	29.1 ± 8.5	21/23	YG	NI	1	6	HADS-D	HADS-A
Nyer et al. (2025) (34)	America	32.7 ± 11.7	33/32	YG	NI	2	8	HDRS-17	STAI
M J Sangeethalaxmi et al. (2023) (35)	India	24.2 ± 2.6	30/30	YG	NI	7	12	BDI	HAM-A
Sanchez-Polan Met al. (2026) (36)	Spain	34.1 ± 4.5	247/244	CE	NI	3	24	EPDS	STAI
Roche LT et al. (2016) (37)	Spain	53.3 ± 11.1	16/22	YG	NI	2	4	BDI-II	BAI
Goran K et al. (2018) (38)	multinational	34.2 ± 4.5	15/15	YG	NI	2	8	SDS	SAS
Karl M F et al. (2021) (39)	Ireland	47.1 ± 10.0	39/41	PL	NI	2	8	HADS-D	HADS-A
Kowk J et al. (2025) (40)	Hong Kong, China	64.8 ± 7.9	52/54	YG	NI	1	8	HADS-D	HADS-A
Tiffany F et al. (2013) (41)	America	26.6 ± 5.5	37/38	TC	NI	1	12	CES-D	STAI
Kyle D et al. (2015) (42)	America	30.2 ± 4.9	23/23	YG	NI	1	8	EPDS	STAI
Cramer et al. (2015) (43)	Germany	49.2 ± 5.9	19/21	YG	NI	1	12	HADS-D	HADS-A
Ciccolo et al. (2022) (44)	America	41.3 ± 12.3	25/25	RE	NI	2	12	PHQ-9	GAD-7
Angela G et al. (2020) (45)	America	29.4 ± 6.9	48/31	YG	NI	2	4	HADS-D	HADS-A
Xu Q et al. (2025) (46)	China	71.2 ± 6.8	44/46	YG	NI	7	8	HAM-D	HAM-A
Kwok J et al. (2019) (47)	Hong Kong, China	63.7 ± 8.7	71/67	YG	NI	2	8	HADS-D	HADS-A

T, Experimental group; C, Control group. TC, Tai Chi; YG, Yoga; MICT, Moderate-intensity continuous training; HIIT, High-intensity interval training; RE, Resistance exercise; AE, Aerobic exercise; CE, Combined exercise; PL, Pilates; NI, No intervention. SDS, Self-rated Depression Scale; SAS, Self-rated Anxiety Scale; DASS-21, Depression-Anxiety-Sociality Scale; BDI, Beck Depression Inventory; GAD-7, Generalized Anxiety Disorder Scale; HAM-D, Hamilton Depression Scale; CES-D, Depression Scale of the Epidemiological Center; STAI, State-Trait Anxiety Inventory; HAM-A, Hamilton Anxiety Scale; HADS-D, Hospital Anxiety and Depression Scale; HADS-A, Hospital Anxiety and Depression Scale-Anxiety Component; HDRS-17,17-item Hamilton Depression Scale; EPDS, Edinburgh Postpartum Depression Scale; BDI-II, Beck Depression Inventory, Second Edition; BAI, Beck Anxiety Inventory; PHQ-9, Patient Health Questionnaire-Depression Module.

### 3.3 Assessment results of bias risk

**Figure 2:**
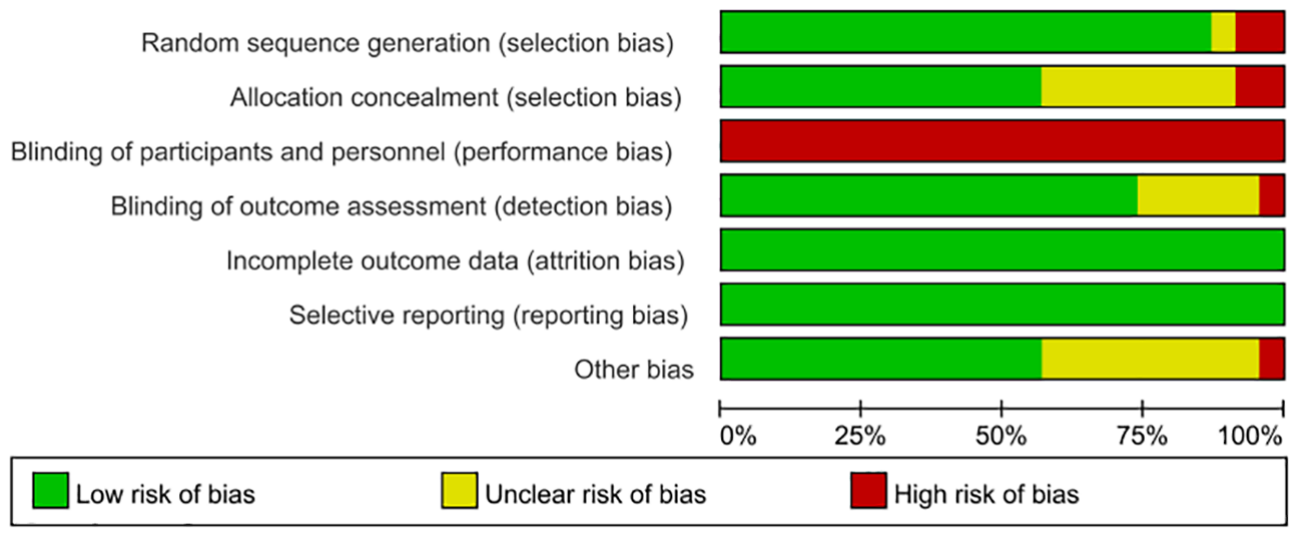
Summary of bias risk.

**Figure 3:**
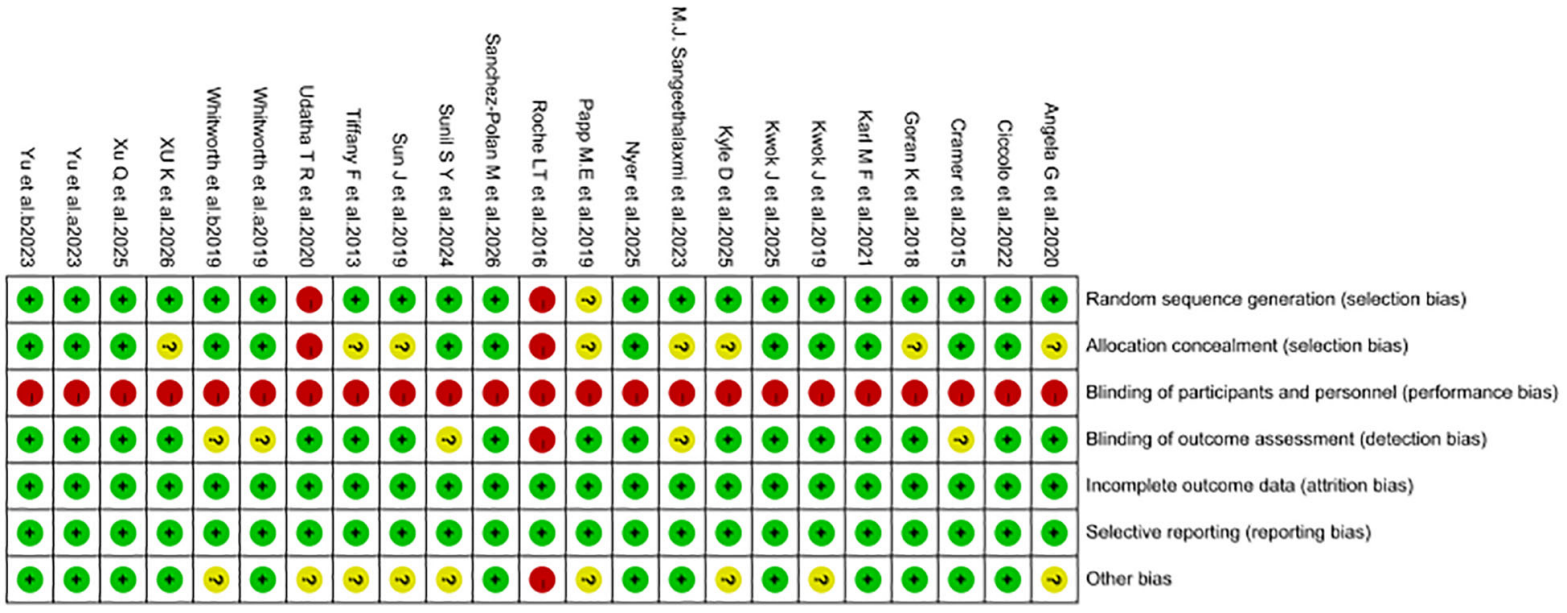
Percentage chart of bias risk.

The Cochrane Rev. 3 tool evaluation results indicated **Figures 2**, **3** that 20 studies (87.0%) were classified as low-risk regarding randomization sequence generation, while 13 studies (56.5%) were low-risk for allocation concealment. Due to the nature of the interventions, all studies (100%) were classified as high-risk in the “blinding of participants and investigators” domain. However, in the more critical “blinding of outcome assessors” domain, 17 studies (73.9%) explicitly reported blinding of scale scorers, thus being classified as low-risk. In terms of data completeness (100% low-risk) and selective reporting (100% low-risk), the overall quality of the included studies was high. Overall, the risk of bias in the included studies was moderate; although bias could not be entirely avoided, the robust blinding of assessors and data completeness ensured that the evidence quality was adequate to support subsequent analyses.

### 3.4 Results of the network meta-analysis

#### 3.4.1 Evidence network structure and contribution degree

This study constructed an evidence network comprising nine intervention nodes: No Intervention Control (NI), Yoga (YG), Tai Chi (TC), Pilates (PL), Resistance Exercise (RE), Aerobic Exercise (AE), Composite Exercise (CE), High-Intensity Interval Training (HIIT), and Moderate-Intensity Continuous Training (MICT).

**Figure 4:**
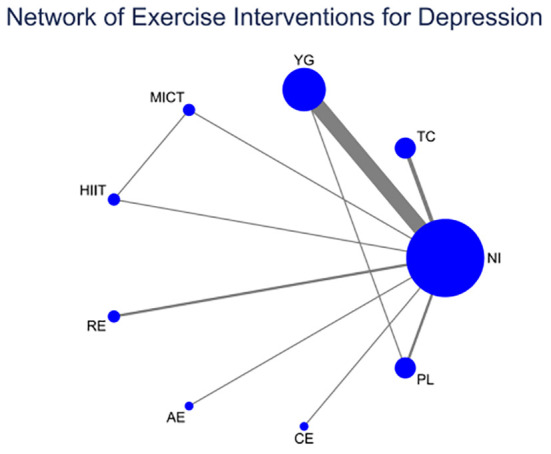
Evidence network map of intervention for depression symptoms.

**Figure 5:**
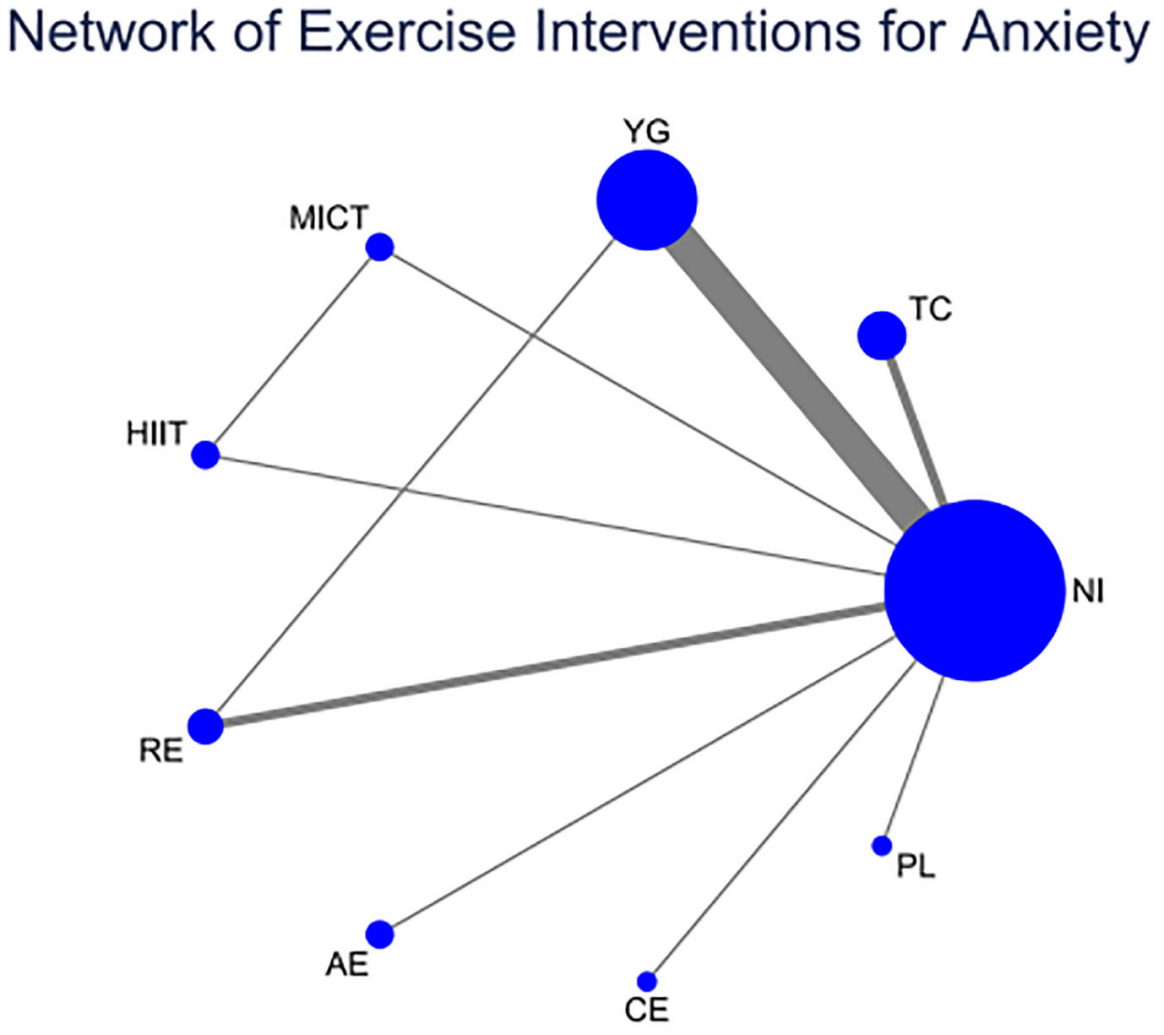
Evidence network map of anxiety symptom intervention.

The evidence network structure for depression and anxiety symptoms (**Figures 4**, **5**) demonstrates that the network centers around the NI, with all movement patterns exhibiting direct comparative evidence relative to the NI. Additionally, a closed loop of direct comparison exists between YG and RE, while comparisons among other nodes primarily rely on indirect evidence. Evidence contribution analysis reveals that the contribution percentages of each comparison group to the overall network are uniformly distributed between 5.0% and 14.5%, with no single study disproportionately dominating the network estimates, indicating a stable and reliable evidence structure. The distribution of effect modification factors for intervention nodes is presented in **Supplementary Table 2**; overall, the comparability among nodes is acceptable.

#### 3.4.2 Intervention effect on depressive symptoms

**Figure 6:**
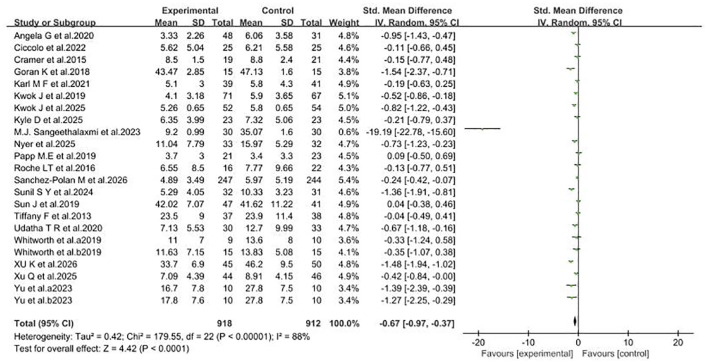
Forest plot of traditional meta-analysis on depressive symptoms.

**Figure 7:**
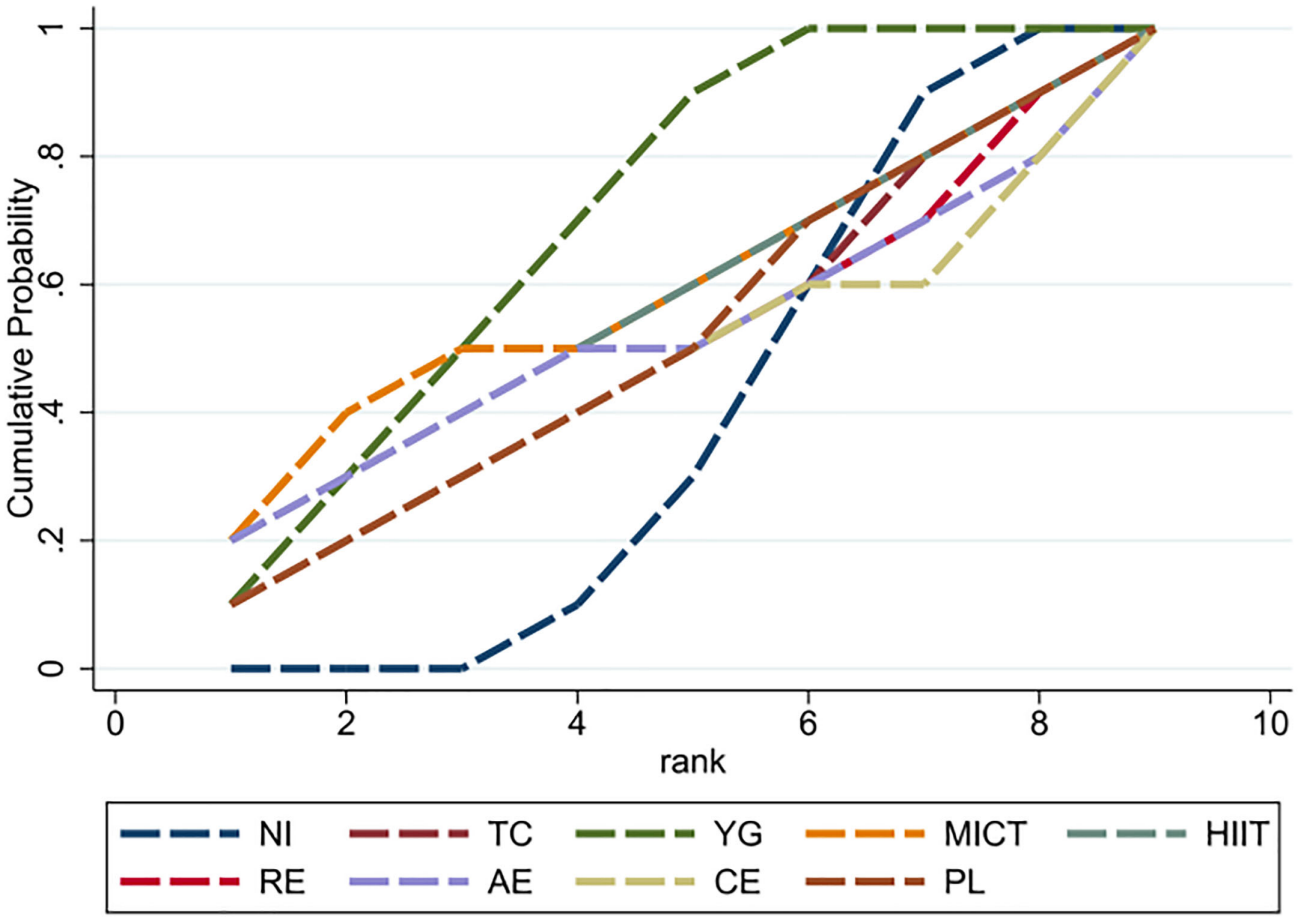
Cumulative ranking probability chart of depression symptoms (SUCRA chart).

Traditional Meta-analysis: A total of 23 RCTs were included. The pooled results from the random effects model showed that the depression scores in the exercise intervention group improved significantly more than those in the control group (SMD = -0.67,95% CI: -0.97 to-0.37, P <0.001), but there was high heterogeneity among the studies (I² = 88%, P <0.001; see **Figure 6**). Network Meta-analysis: The league table (**Table 2**) indicated that the SMD estimates for all eight exercise modalities were negative, suggesting superior efficacy in improving depressive symptoms compared to the NI group. Among these, YG, MICT, and HIIT demonstrated the largest effect sizes. SUCRA Ranking: The cumulative ranking probability plot (**Figure 7**) showed that yoga had the highest probability of being the optimal intervention (SUCRA = 68.8%), followed by MICT (56.7%), HIIT (54.6%), aerobic exercise (50.0%), Pilates (47.1%), Tai Chi (46.6%), combined exercise (44.8%), resistance exercise (44.5%), and NI (36.9%). For details, see **Table 3**. It is important to note that although yoga ranked first in the SUCRA, the pairwise comparisons between yoga and MICT or HIIT were not statistically significant; therefore, this ranking should be interpreted as a probabilistic priority rather than evidence of superiority.

**Table 2:** League table of network meta-analysis on depression symptoms.

Variable	NI	PL	CE	AE	RE	HIIT	MICT	YG	TC
NI	–								
PL	0.58 (-3.88,5.03)	–							
CE	0.24 (-7.36,7.84)	-0.33 (-9.14,8.48)	–						
AE	0.67 (-6.94,8.29)	0.10 (-8.73,8.92)	0.43 (-10.33,11.19)	–					
RE	0.34 (-5.07,5.74)	-0.24 (-7.24,6.77)	0.09 (-9.23,9.42)	-0.34 (-9.67,9.00)	–				
HIIT	1.27 (-6.38,8.93)	0.70 (-8.16,9.56)	1.03 (-9.76,11.82)	0.60 (-10.20,11.40)	0.94 (-8.43,10.31)	–			
MICT	1.41 (-6.24,9.07)	0.84 (-8.02,9.70)	1.17 (-9.62,11.96)	0.74 (-10.06,11.54)	1.08 (-8.30,10.45)	0.14 (-7.51,7.79)	–		
YG	1.95 (-0.30,4.21)	1.38 (-3.27,6.02)	1.71 (-6.22,9.64)	1.28 (-6.66,9.22)	1.62 (-4.24,7.47)	0.68 (-7.30,8.66)	0.54 (-7.44,8.52)	–	
TC	0.49 (-3.90,4.89)	-0.08 (-6.34,6.18)	0.25 (-8.53,9.03)	-0.18 (-8.97,8.61)	0.15 (-6.81,7.12)	-0.78 (-9.61,8.05)	-0.92 (-9.75,7.91)	-1.46 (-6.40,3.48)	–

**Table 3:** SUCRA probability ranking of depressive and anxiety symptom outcomes.

Intervene	Depressed	Rank	Anxious	Rank
	sucra		sucra	
YG	68.8	1	72.2	1
MICT	56.7	2	56.2	3
HIIT	54.6	3	57.4	2
AE	50.0	4	45.7	6
PL	47.1	5	44.5	7
TC	46.6	6	46.6	5
CE	44.8	7	43.0	8
RE	44.5	8	48.0	4
NI	36.9	9	36.5	9

#### 3.4.3 Intervention effect on anxiety symptoms

**Figure 8:**
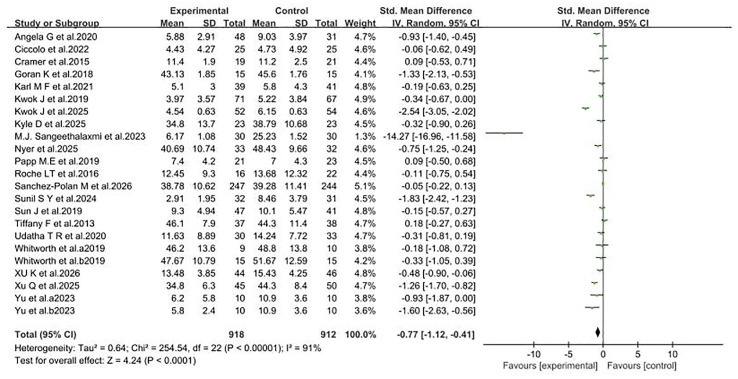
Forest plot of traditional meta-analysis on anxiety symptoms.

**Figure 9:**
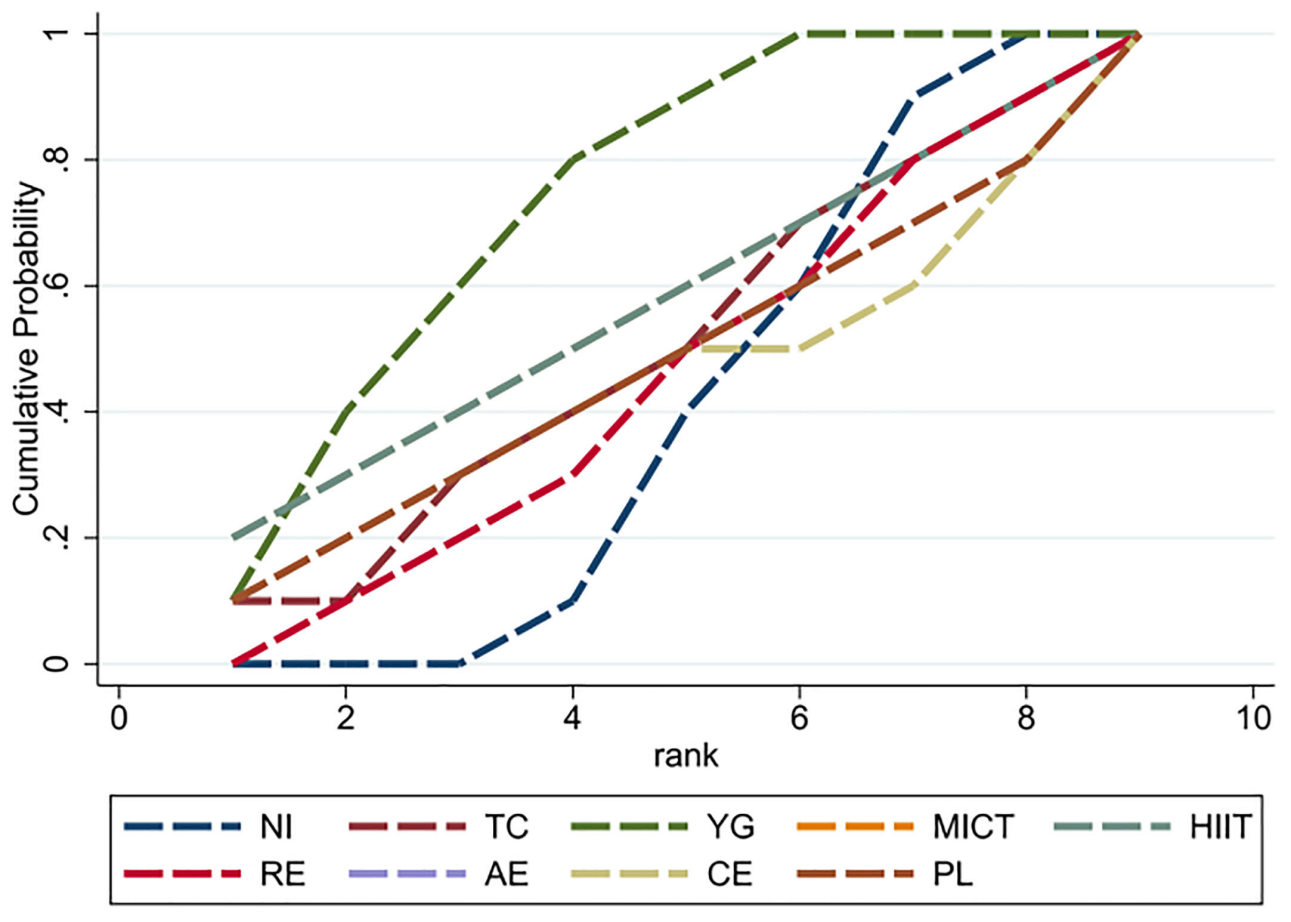
Cumulative ranking probability chart of anxiety symptoms (SUCRA chart).

Traditional Meta-analysis: The pooled results from the random-effects model demonstrated that the exercise intervention group showed significantly better improvement in anxiety scores compared to the control group (SMD = -0.77,95% CI: -1.12 to-0.41, P <0.001), with high heterogeneity among studies (I² = 91%, P <0.001; see **Figure 8**). Network Meta-analysis: The league table (**Table 4**) indicated that all eight exercise modalities exhibited superior anxiety improvement effects compared to NI. Yoga (YG), HIIT, and MICT demonstrated relatively larger effect sizes. SUCRA Ranking: The cumulative ranking probability plot (**Figure 9**) showed that yoga had the highest probability of being the optimal intervention (SUCRA = 72.2%), followed by HIIT (57.4%), MICT (56.2%), resistance exercise (48.0%), Tai Chi (46.6%), aerobic exercise (45.7%), Pilates (44.5%), combined exercise (43.0%), and NI (36.5%). For details, see **Table 3**. Similar to the depression results, the differences between yoga and HIIT/MICT were not statistically significant; thus, the rankings should be interpreted exploratorily.

**Table 4:** League table of network meta-analysis on anxiety symptoms.

Variable	NI	PL	CE	AE	RE	HIIT	MICT	YG	TC
NI	–								
PL	0.19 (-5.47,5.85)	–							
CE	0.05 (-5.60,5.69)	-0.14 (-8.14,7.85)	–						
AE	0.31 (-5.35,5.98)	0.12 (-7.88,8.13)	0.27 (-7.73,8.26)	–					
RE	0.50 (-2.37,3.37)	0.31 (-6.03,6.66)	0.45 (-5.88,6.79)	0.19 (-6.16,6.54)	–				
HIIT	1.19 (-4.53,6.91)	1.00 (-7.05,9.05)	1.14 (-6.90,9.18)	0.88 (-7.17,8.93)	0.69 (-5.71,7.09)	–			
MICT	1.09 (-4.63,6.81)	0.91 (-7.14,8.95)	1.05 (-6.99,9.08)	0.78 (-7.27,8.83)	0.59 (-5.81,6.99)	-0.09 (-5.80,5.62)	–		
YG	1.75 (0.08,3.42)	1.56 (-4.34,7.46)	1.70 (-4.19,7.59)	1.44 (-4.47,7.34)	1.25 (-1.85,4.35)	0.56 (-5.40,6.52)	0.66 (-5.30,6.61)	–	
TC	0.41 (-2.86,3.68)	0.22 (-6.31,6.76)	0.37 (-6.16,6.89)	0.10 (-6.44,6.64)	-0.09 (-4.44,4.26)	-0.78 (-7.36,5.81)	-0.68 (-7.27,5.90)	-1.34 (-5.01,2.33)	–

#### 3.4.4 Consistency test

**Figure 10:**
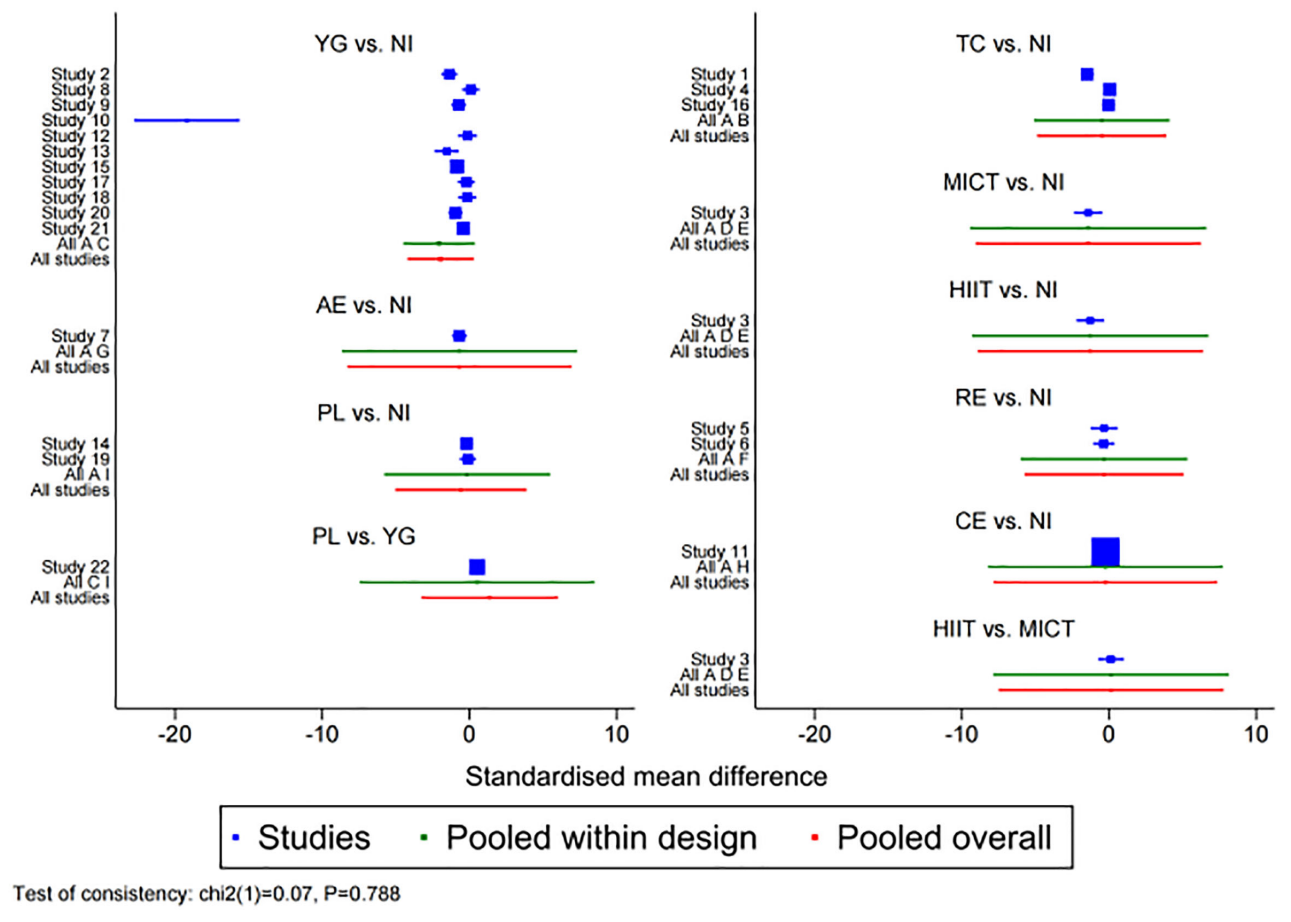
Forest plot of consistency test for the node splitting method of depressive symptoms.

**Figure 11:**
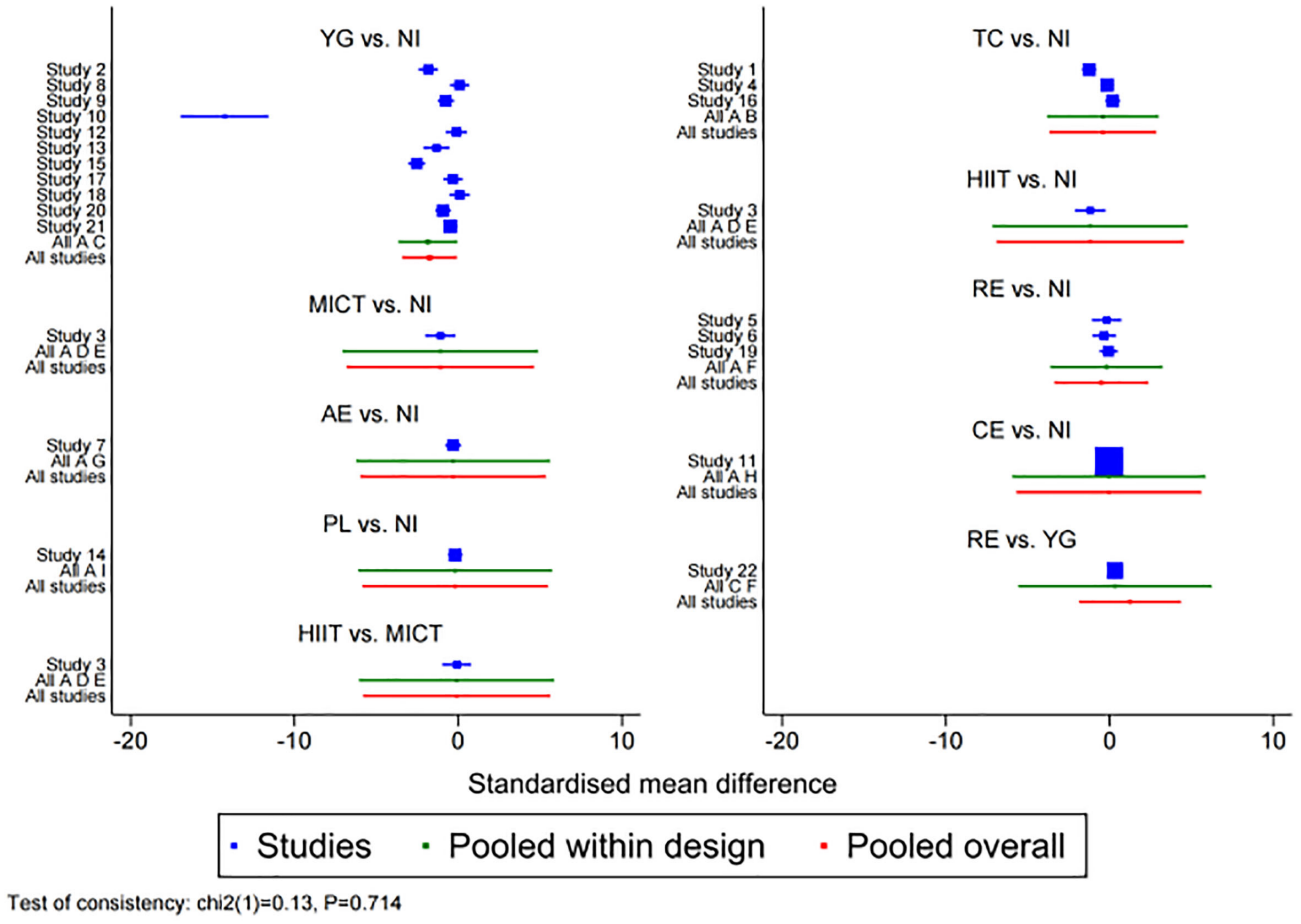
Forest plot of consistency test for the node splitting method of anxiety symptoms.

The consistency test for the closed loop of YG vs RE was performed using the node splitting method (**Figures 10**, **11**). The depression symptom network yielded a χ² value of 0.07 (P = 0.788), while the anxiety symptom network yielded a χ² value of 0.13 (P = 0.714). Both P-values were greater than 0.05, indicating no statistically significant difference between the direct and indirect evidence comparisons. The overall consistency of the evidence network is strong, supporting the use of the consistency model for NMA.

#### 3.4.5 Subgroup analysis results

##### 3.4.5.1 Subgroup analysis of depressive symptoms

**Figure 12:**
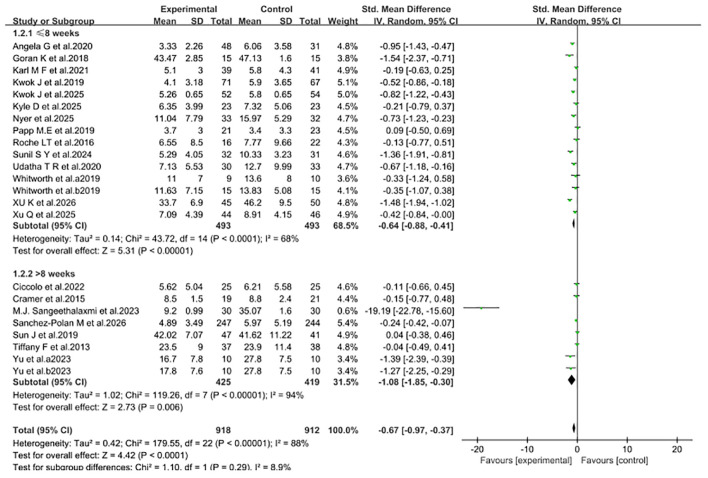
Forest plot showing stratified subgroup analysis of depressive symptoms by intervention cycle.

Stratification by intervention duration (**Figure 12**): ≤8 weeks subgroup: included 15 studies, with 493 cases in the experimental group and 493 cases in the control group; pooled effect size SMD = -0.64,95% CI (-0.88, -0.41), P <0.00001, I² = 68%;>8 weeks subgroup: included 8 studies, with 425 cases in the experimental group and 419 cases in the control group; pooled effect size SMD = -1.08,95% CI (-1.85, -0.30), P = 0.006, I² = 94%. Subgroup comparison test: χ² = 1.10, df = 1, P = 0.29, indicating that intervention duration was not a significant moderating factor for the depressive intervention effect.

**Figure 13:**
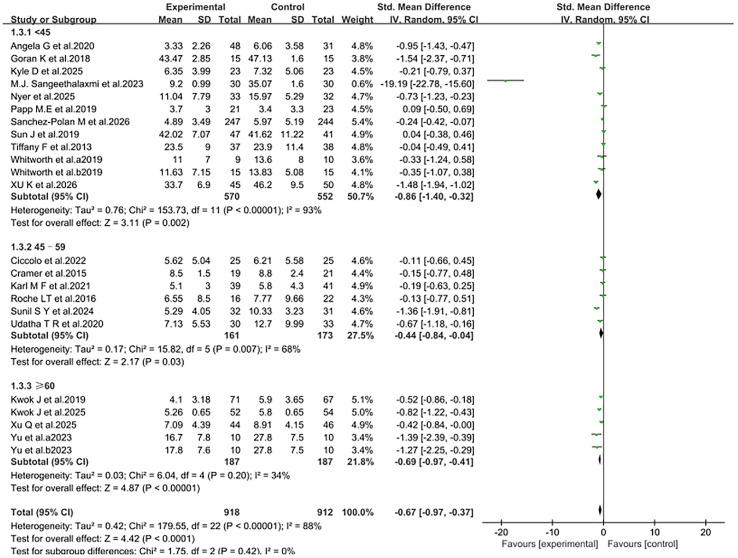
Forest plot showing age-stratified subgroup analysis of depressive symptoms.

Age-stratified analysis (**Figure 13**): In the ≤45-year-old subgroup, 12 studies were included, with 570 cases in the experimental group and 552 cases in the control group. The pooled effect size (SMD) was −0.86,95% CI (-1.40, −0.32), P = 0.002, I² = 93%. In the 45–59-year-old subgroup, 6 studies were included, with 161 cases in the experimental group and 173 cases in the control group. The pooled SMD was −0.44,95% CI (-0.84, −0.04), P = 0.03, I² = 68%. In the ≥60-year-old subgroup, 5 studies were included, with 187 cases in both the experimental and control groups. The pooled SMD was −1.27,95% CI (-2.25, −0.29), P = 0.01, I² = 34%. Subgroup comparison test: χ² = 1.75, df = 2, P = 0.42, indicating that age was not a significant moderating factor for the depressive intervention effect.

##### 3.4.5.2 Subgroup analysis of anxiety symptoms

**Figure 14:**
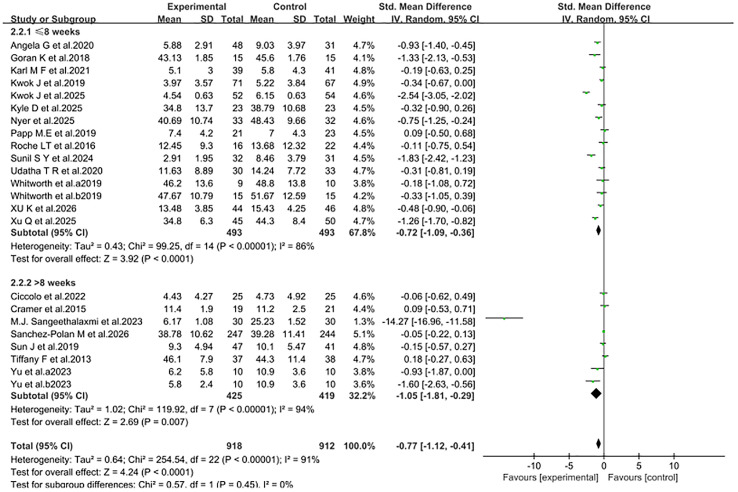
Forest plot showing stratified subgroup analysis of anxiety symptoms by intervention cycle.

Stratification by intervention duration (**Figure 14**): ≤8 weeks subgroup: included 15 studies, with 493 cases in the experimental group and 493 cases in the control group; pooled effect size SMD = -0.72,95% CI (-1.09, -0.36), P <0.0001, I² = 86%;>8 weeks subgroup: included 8 studies, with 425 cases in the experimental group and 419 cases in the control group; pooled effect size SMD = -1.05,95% CI (-1.81, -0.29), P = 0.007, I² = 94%. Subgroup comparison test: χ² = 0.57, df = 1, P = 0.45, indicating that intervention duration was not a significant moderating factor for the anxiety intervention effect.

**Figure 15:**
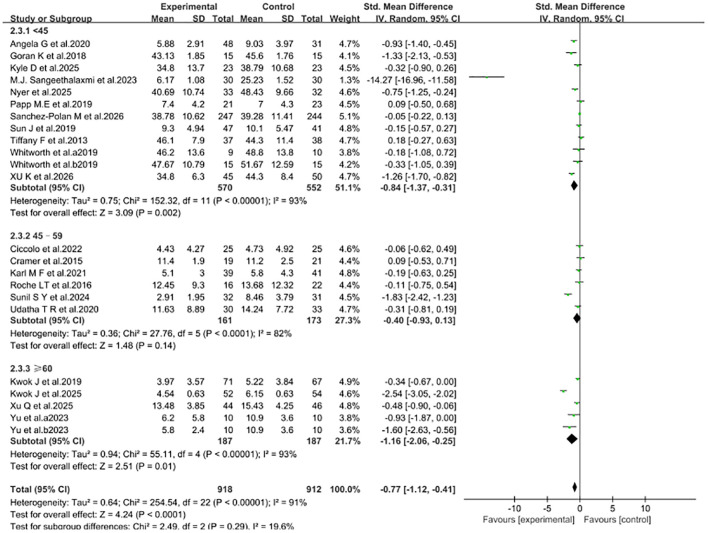
Forest plot of age-stratified subgroup analysis of anxiety symptoms.

Age-stratified analysis (**Figure 15**): ≤45-year-old subgroup: Involving 12 studies, with 570 cases in the experimental group and 552 cases in the control group; pooled effect size (SMD) = -0.84,95% CI (-1.37, -0.31), P = 0.002, I² = 93%.45–59-year-old subgroup: Involving 6 studies, with 161 cases in the experimental group and 173 cases in the control group; pooled effect size (SMD) = -0.40,95% CI (-0.93,0.13), P = 0.14, I² = 82%. ≥60-year-old subgroup: Involving 5 studies, with 187 cases in the experimental group and 187 cases in the control group; pooled effect size (SMD) = -1.16,95% CI (-2.06, -0.25), P = 0.01, I² = 93%. Subgroup comparison test: χ² = 2.49, df = 2, P = 0.29, indicating that age was not a significant moderating factor for the anxiety intervention effect.

#### 3.4.6 Publication bias assessment, meta-regression analysis, and correction

**Figure 16:**
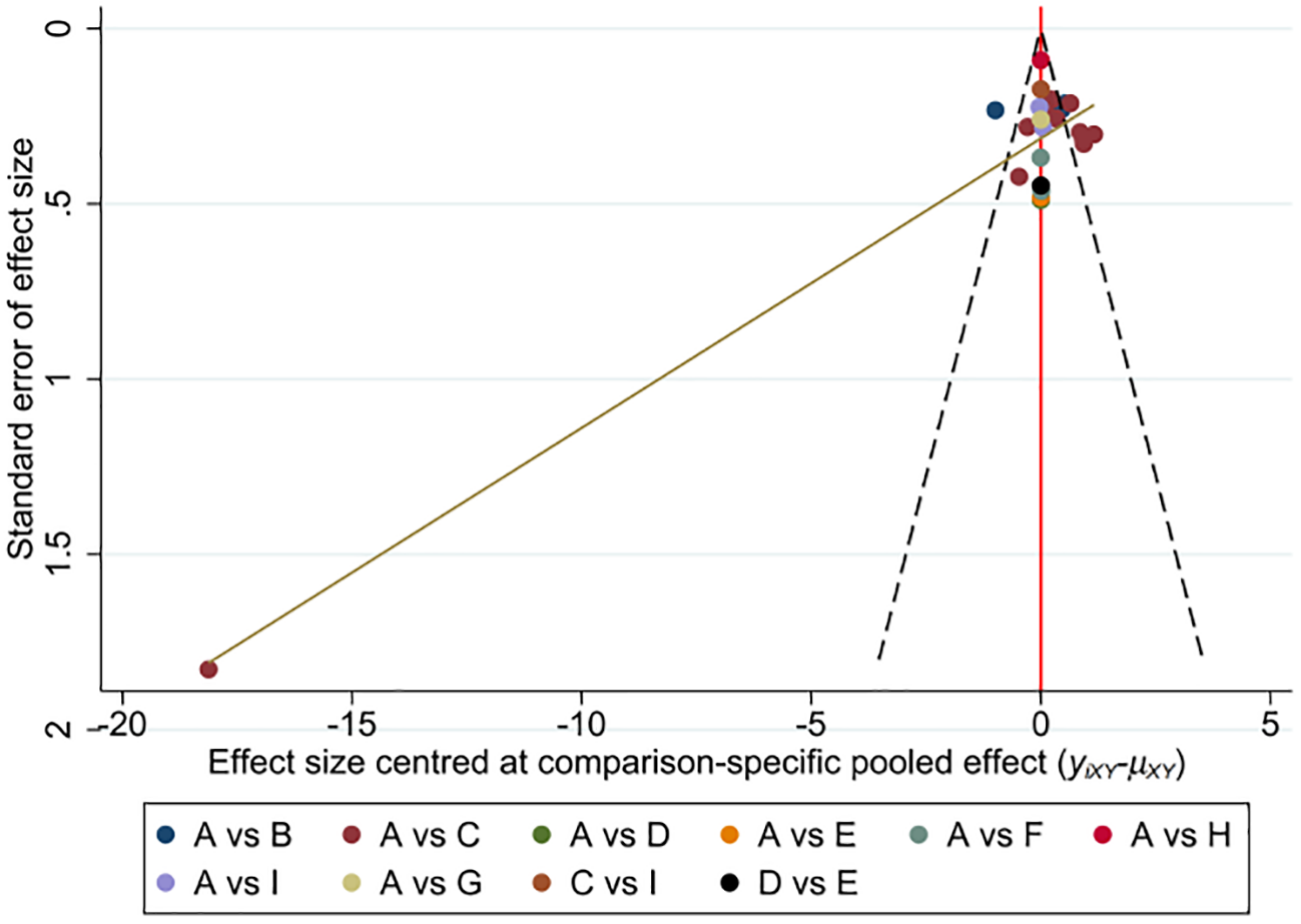
Depression symptom correction funnel chart.

**Figure 17:**
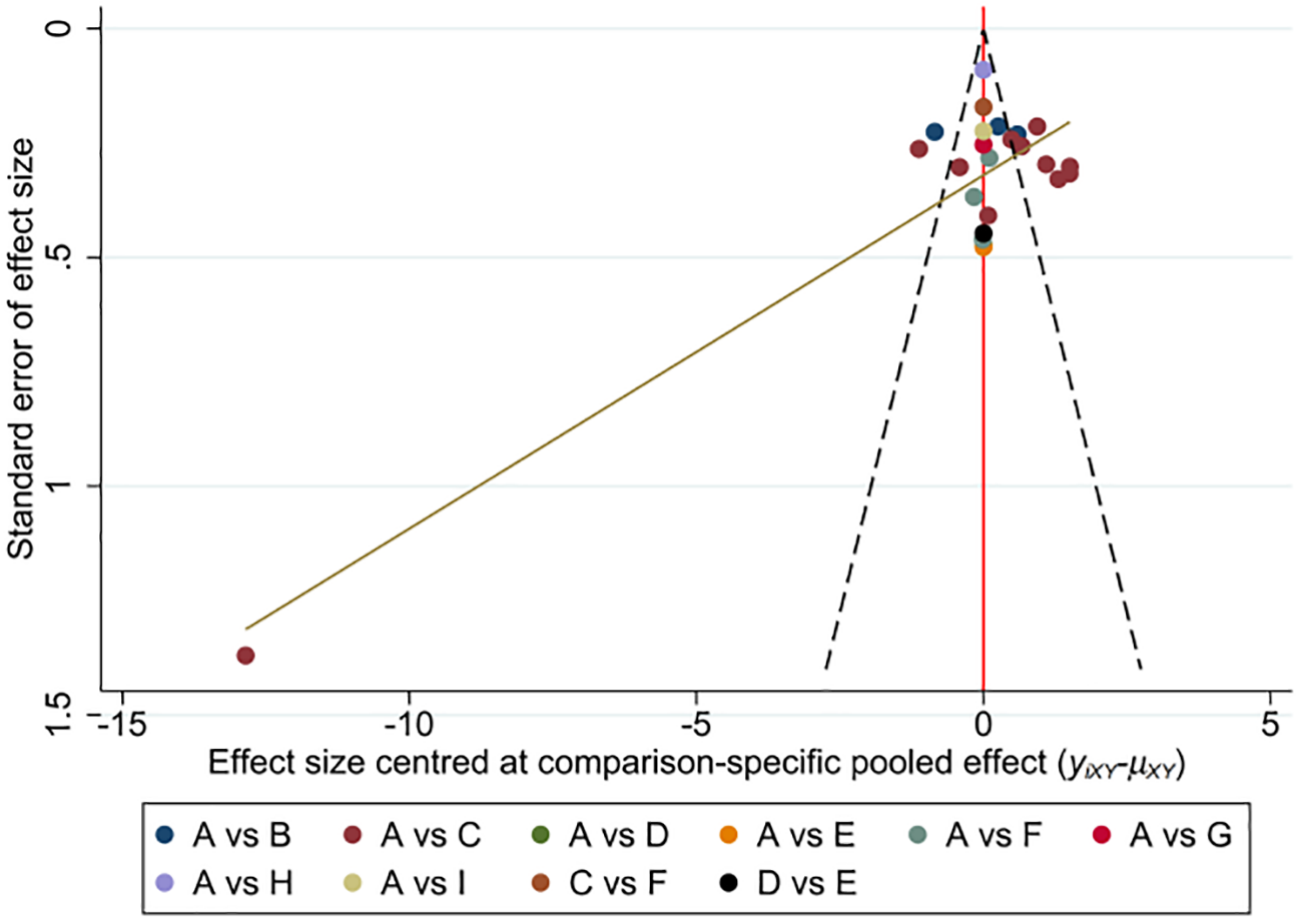
Anxiety symptom correction funnel diagram.

The publication bias for depression and anxiety outcomes was assessed using the corrected funnel plot (**Figures 16**, **17**), Begg’s test, and Egger’s test, respectively. For depressive symptoms: the Begg’s test (after continuity correction) yielded P = 0.097> 0.05, indicating no statistical significance; however, the Egger’s test revealed a bias term P = 0.017 <0.05, suggesting potential publication bias. For anxiety symptoms: the Begg’s test (after continuity correction) yielded P = 0.035 <0.05, and the Egger’s test showed a bias term P = 0.010 <0.05, also indicating possible publication bias.

To further evaluate the impact of publication bias on the pooled effect size, a non-parametric trim-and-fill method was employed for correction analysis. The results demonstrated that neither depression nor anxiety outcomes required any imputation of missing studies (imputed=0). The pooled effect sizes and 95% confidence intervals remained unchanged before and after correction.

To thoroughly investigate the sources of heterogeneity and evaluate the small-sample effect, this study further employed a random-effects meta-regression analysis, using the standard error of the effect size (_seES) as a covariate to examine its significant impact on the pooled effect size (_ES). The meta-regression analysis for depressive symptoms revealed no statistically significant effect of this covariate (P = 0.928), indicating the absence of a pronounced small-sample effect. Similarly, the meta-regression results for anxiety symptoms also showed no small-sample effect (P = 0.938). It should be noted that the R² values in the meta-regressions were exceptionally high, which may be attributed to the limited number of included studies and the risk of model overfitting; therefore, caution should be exercised when interpreting the extent of heterogeneity explained.

Based on the above findings: The Egger’s test suggests possible publication bias, but the imputation method did not address missing studies, and meta-regression did not reveal small-sample effects. This indicates that the asymmetry in the funnel plot may partly stem from clinical heterogeneity among studies (e.g., intervention protocols, population characteristics) rather than pure publication bias. Consequently, the pooled effect size of this study is generally robust, and the conclusions are credible; however, the potential impact of publication bias cannot be entirely ruled out.

#### 3.4.7 Sensitivity analysis

**Figure 18:**
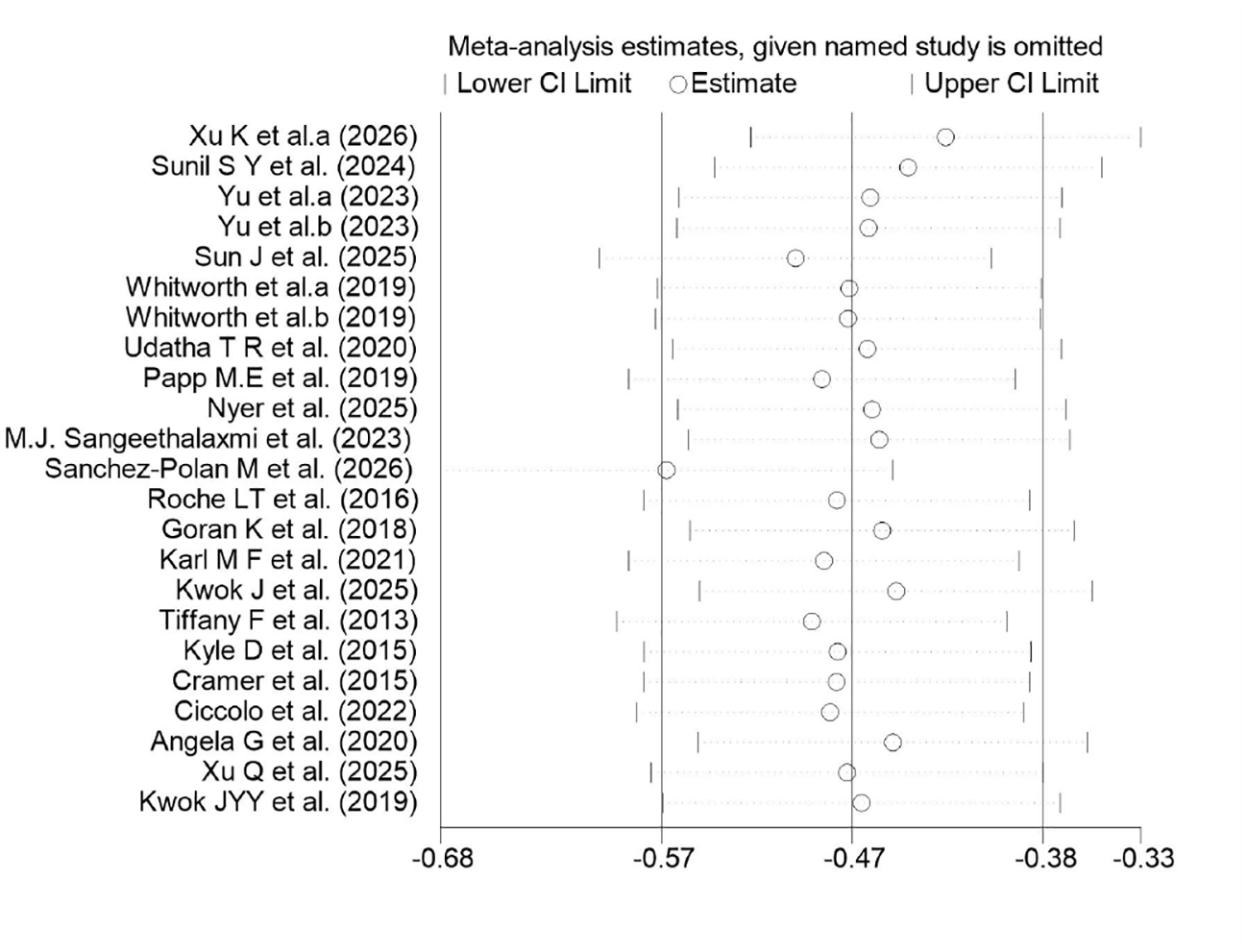
Sensitivity analysis plot after excluding individual studies (depressive outcomes).

**Figure 19:**
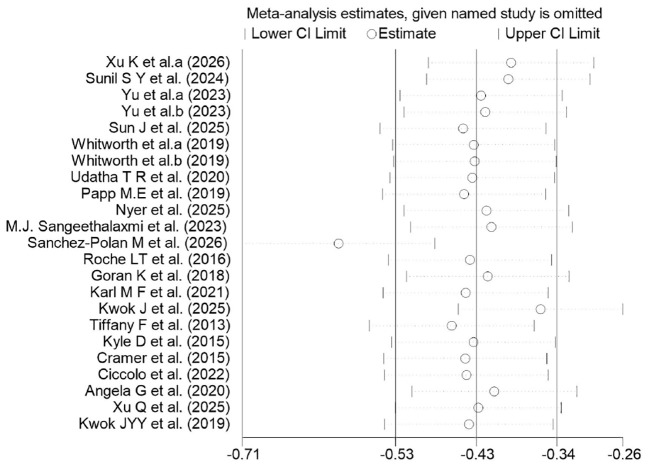
Sensitivity analysis plot after excluding individual studies (anxiety outcome).

The sensitivity analysis of the combined effect sizes for depressive and anxiety symptoms was conducted using the sequential exclusion method for individual studies. The results demonstrated that after excluding any single study, neither the point estimate nor the 95% confidence interval of the combined effect size (SMD) for depressive symptoms underwent a change in direction, with the effect size fluctuating between-0.57 and-0.47; similarly, the combined effect size for anxiety symptoms ranged from-0.53 to-0.34. All results remained within the consistent direction of the overall effect, without any reversal of conclusions driven by a single study (**Figures 18**, **19**). Notably, after excluding Sanchez-Polan et al. (2026), the combined effect size for anxiety symptoms exhibited the largest fluctuation (SMD = -0.60), yet this result still aligned with the overall direction of the combined effect and did not overturn the conclusions. Overall, the meta-analysis results for both depressive and anxiety symptoms demonstrated robust validity, unaffected by individual studies.

## 4 Discussion

### 4.1 Overall effects and sources of heterogeneity of exercise intervention

The results of this traditional meta-analysis are highly consistent with those of previous large-scale reviews, confirming the central role of exercise as an effective intervention for mood disorders. However, significant heterogeneity was observed among the studies (I²> 88%,91%). The sources of heterogeneity included not only differences in exercise protocol parameters (frequency, duration) and assessment tools (self-report vs. other-report), but also variations in participants ‘clinical characteristics (diagnostic criteria, symptom severity, age range). The exercise effects may differ between specific disease populations (e.g., Parkinson’s disease) and the general population (48). Additionally, approximately 40% of the included studies had small sample sizes (n <30), yet the meta-regression analysis did not reveal any small-sample effects, indicating that the differences in effect sizes primarily stemmed from genuine intervention effects rather than sample size limitations. Future studies should define study populations more rigorously and employ meta-analyses of individual participant data to investigate the sources of heterogeneity.

### 4.2 Therapeutic specificity and neurobiological mechanisms of different exercise modes

Depressive symptoms: Performance of yoga, MICT, and HIIT. This study found that yoga ranked first in the SUCRA probability ranking, followed closely by MICT and HIIT. However, pairwise comparisons revealed no statistically significant differences in effect sizes between yoga and MICT or HIIT. Therefore, yoga, MICT, and HIIT can all be considered preferred exercise modalities for improving depression (49, 50). The antidepressant mechanisms of yoga may involve downregulating excessive activation of the hypothalamic-pituitary-adrenal axis, enhancing prefrontal emotional regulation of the amygdala, and increasing heart rate variability (51–53); some evidence suggests that autonomic nervous system modulation may play a role (19). MICT promotes stable release of brain-derived neurotrophic factors and balance of monoamine neurotransmitters through sustained moderate-intensity exercise (50, 54). Although HIIT involves shorter single-session durations, its high-intensity intermittent stimulation induces stronger increases in BDNF expression and activation of the endogenous cannabinoid system (55, 56). These neurobiological mechanisms require further direct evidence for confirmation.

Anxiety symptoms: Yoga and HIIT demonstrated the most pronounced effects. In anxiety intervention studies, yoga ranked first in the SUCRA scale, followed by HIIT, though the difference between the two was also non statistically significant. Yoga activates the parasympathetic nervous system through rhythmic breathing and slow movements, thereby reducing physiological arousal levels (57). Potential mechanisms of HIIT in anti-anxiety effects include the “post-stress relaxation response” induced by high-intensity exercise and the enhanced prefrontal regulatory function mediated by intermittent hypoxia-reoxygenation (58). Although HIIT ranked slightly higher than MICT in the SUCRA scale, the difference in their SMD values was not statistically significant; therefore, it cannot be concluded that HIIT exhibits superior anti-anxiety effects compared to MICT.

### 4.3 Value and clinical application implications of compound movements

Composite exercise regimens maintain a mid-ranking position in both outcomes. From a mechanistic perspective, integrated programs combining aerobic, resistance, or mind-body elements may synergistically deliver the physiological and psychological benefits of multiple exercises, particularly for patients with comorbid depression and anxiety. Given the absence of statistically significant differences among yoga, MICT, and HIIT, clinical decision-making should comprehensively consider patient preferences, symptom profiles, baseline physical fitness, tolerability, and safety profiles rather than relying solely on the SUCRA rankings. Current evidence is insufficient to achieve optimal matching at the symptom cluster level; a shared decision-making approach is recommended. For patients dominated by depressive symptoms (e.g., anhedonia, fatigue, low self-esteem), yoga, MICT, or HIIT should be prioritized; for those dominated by anxiety symptoms (e.g., excessive worry, somatic tension), mind-body exercises such as yoga or Tai Chi, along with tolerable HIIT, are viable options; for comorbid patients, well-designed composite exercise regimens may represent the optimal strategy (59, 60).

In clinical translation, feasibility must also be carefully considered. Depression and anxiety are often accompanied by low motivation, fatigue, and difficulties in accessing monitoring tools, which may reduce exercise adherence. Digital or home-based exercise programs may help overcome some of these barriers; however, existing evidence remains inconsistent, and high-quality studies are still needed to validate their efficacy and sustainability (61). When recommending high-intensity exercise regimens, medical screening is particularly essential to ensure safety.

### 4.4 Discussion on the robustness and sources of heterogeneity of the results

The traditional meta-analyses of depressive and anxiety outcomes in this study all demonstrated high heterogeneity. To identify the sources of heterogeneity and validate the reliability of the network meta-analysis results, this study conducted systematic robustness assessments across multiple dimensions, including consistency testing, subgroup analysis, meta-regression, and publication bias correction. The consistency testing revealed a high concordance between direct and indirect evidence. Subgroup analysis indicated that the moderating effects of age and intervention duration were not statistically significant; however, descriptive comparisons suggested that the effect size might be larger in the elderly cohort, a trend warranting further investigation. Meta-regression analysis demonstrated that the constructed model accounted for the majority of heterogeneity, with no small-sample effects identified. Regarding publication bias, Egger’s test suggested a potential presence of publication bias; however, the method did not incorporate missing studies, and sensitivity analyses yielded stable results, indicating that the conclusions are generally robust. Nevertheless, the potential risk of publication bias should not be entirely ruled out.

### 4.5 Research advantages, limitations, and future directions

Advantages: This study is the first to conduct a comparative analysis and ranking of eight refined structured movement patterns in adults using NMA. The methodological process was rigorous, encompassing registration in PROSPERO, adherence to PRISMA-NMA guidelines, stringent assessment of bias risks, consistency testing and publication bias correction, and meta-regression analysis to evaluate small-sample effects. Sensitivity analyses confirmed the robustness of the conclusions.

Limitations: ①Despite the use of a random effects model, high heterogeneity among studies still affects the precision of combined effect sizes. ②A limited number of studies (n ≤ 2) were included for certain exercise modalities (e.g., aerobic exercise, HIIT, MICT), and the corresponding SUCRA rankings may be revised as more evidence becomes available; Pilates was assessed in only one RCT, and its independent effect remains unverified. ③Due to constraints imposed by the original study data, detailed subgroup analyses or meta-regressions regarding age, gender, baseline symptom severity, and exercise dosage were not conducted, limiting the accuracy of personalized recommendations. ④The included studies primarily focused on short-term interventions (median duration: 8 weeks), lacking long-term follow-up data, leaving the sustained effects of exercise interventions unclear. ⑤Although adjustments and meta-regression analyses were performed, the potential impacts of publication bias and small-sample effects cannot be entirely ruled out. ⑥Some included studies targeted specific disease populations (e.g., Parkinson’s disease); although the need for interventions targeting depressive/anxious symptoms is equally urgent in these populations, their exercise effects and acceptability may differ from those in the general population, and this clinical heterogeneity must be considered when generalizing findings.

Future Directions: More high-quality, head-to-head randomized controlled trials (RCTs) should be conducted, particularly for exercise modalities with weak evidence bases (e.g., HIIT vs MICT). Research should focus on exploring the dose-response relationship of exercise interventions and utilize biomarkers (e.g., BDNF, heart rate variability, cortisol awakening response) to predict intervention efficacy. Future implementation may benefit from digital or AI-assisted tools guided by clinicians to match patients with feasible exercise forms, monitor adherence, and dynamically adjust parameters; however, such tools should support rather than replace clinical judgment, especially when mental symptoms or comorbidities are present, requiring cautious use (62). For individuals with severe fatigue, low motivation, or functional limitations, whether assisted or passive exercise methods can serve as transitional approaches should be rigorously evaluated in independent trials and distinguished from active structured exercise (63). Conducting meta-analyses of individual participant data will help identify subgroups that benefit most from specific exercise modalities. Therefore, future trials should incorporate well-designed direct comparisons, standardized intervention reports, longer follow-up periods, and outcome measures encompassing adherence, safety, acceptability, and maintenance effects.

## 5 Conclusion

This study, based on a network meta-analysis, demonstrated that eight structured exercise modalities—yoga, Tai Chi, resistance training, aerobic exercise, compound exercise, HIIT, MICT, and Pilates—all significantly improved depressive and anxiety symptoms in adults. The therapeutic effects varied across different exercise modalities. In the SUCRA probability ranking, yoga ranked first for both depression and anxiety; however, pairwise comparisons revealed that the differences in efficacy between yoga and MICT or HIIT were not statistically significant, indicating that this ranking is exploratory rather than confirmatory. Yoga, moderate-intensity continuous training (MICT), and high-intensity interval training (HIIT) can all be considered prioritized exercise options for alleviating depression and anxiety. Clinical selection should be based on a comprehensive evaluation of the patient’s dominant symptom clusters, tolerance, preferences, and safety profile. This study provides high-quality evidence-based support for exercise as an adjunctive therapy for mental disorders; however, further head-to-head comparisons and long-term follow-up studies are warranted.

## Data Availability

The raw data supporting the conclusions of this article will be made available by the authors, without undue reservation.
