# Supplementary material for: Comparative effects of structured exercise protocols on depression and anxiety symptoms: a network meta-analysis
Source: Front Psychiatry. 2026 Jun 8;17:1870361. doi: 10.3389/fpsyt.2026.1870361 (PMC13284056; doi:10.3389/fpsyt.2026.1870361)
Supplement: Supplementary file 1 [file Table1.docx]

Web of Science检索策略：

#1 TS=("exercise" OR "exercise therapy" OR aerobic* OR resistance OR "mind-body" OR yoga OR "tai chi" OR pilates OR "high-intensity interval" OR HIIT OR "moderate-intensity continuous" OR MICT)

#2 TS=("depression" OR depress* OR "anxiety" OR anxiety OR "mood disorder")

#3 TS=("randomized controlled trial" OR "controlled clinical trial" OR random* OR RCT)

#4 #1 AND #2 AND #3

Embase检索策略：

#1 'exercise'/exp OR 'kinesiotherapy'/exp OR aerobic*:ti,ab,kw OR resistance:ti,ab,kw OR 'mind-body':ti,ab,kw OR yoga:ti,ab,kw OR 'tai chi':ti,ab,kw OR pilates:ti,ab,kw OR 'high-intensity interval':ti,ab,kw OR hiit:ti,ab,kw OR 'moderate-intensity continuous':ti,ab,kw OR mict:ti,ab,kw

#2 'depression'/exp OR 'anxiety'/exp OR depress*:ti,ab,kw OR anxiety:ti,ab,kw OR 'mood disorder':ti,ab,kw

#3 'randomized controlled trial'/exp OR 'controlled clinical trial'/exp OR random*:ti,ab,kw OR rct:ti,ab,kw

#4 #1 AND #2 AND #3

Cochrane Library检索策略：

#1 'exercise'/exp OR 'kinesiotherapy'/exp OR aerobic*:ti,ab,kw OR resistance:ti,ab,kw OR 'mind-body':ti,ab,kw OR yoga:ti,ab,kw OR 'tai chi':ti,ab,kw OR pilates:ti,ab,kw OR 'high-intensity interval':ti,ab,kw OR hiit:ti,ab,kw OR 'moderate-intensity continuous':ti,ab,kw OR mict:ti,ab,kw

#2 'depression'/exp OR 'anxiety'/exp OR depress*:ti,ab,kw OR anxiety:ti,ab,kw OR 'mood disorder':ti,ab,kw

#3 'randomized controlled trial'/exp OR 'controlled clinical trial'/exp OR random*:ti,ab,kw OR rct:ti,ab,kw

#4 #1 AND #2 AND #3
